# Honey Bee Virus Transmission via Hive Products

**DOI:** 10.3390/vetsci7030096

**Published:** 2020-07-21

**Authors:** Dominik Schittny, Orlando Yañez, Peter Neumann

**Affiliations:** 1 Institute of Bee Health, Vetsuisse Faculty, University of Bern, 3097 Bern, Switzerland; dominik.schittny@ana.unibe.ch (D.S.); peter.neumann@vetsuisse.unibe.ch (P.N.); 2 Agroscope, Swiss Bee Research Center, 3097 Bern, Switzerland

**Keywords:** honey bee, virus, DWV-A, hive products, honey, pollen, wax

## Abstract

The global trade of honey bee hive products has raised concern about pathogen transmission. However, the efficacy of hive products as virus vehicles is poorly understood. Here, we investigated the transmission capacity of hive products for Deformed wing virus genotype A (DWV-A) in a fully-crossed hoarding cage experiment and estimated the transmission risk by screening commercial products. Western honey bee workers were provided with honey, pollen and wax either contaminated with high (~2 × 10^9^), medium (~1.7 × 10^8^), low (~8 × 10^6^) or zero (control) DWV-A genome copies. For 10 days, mortality was monitored. Then, virus titers were quantified in bee heads and 38 commercial products using RT-qPCR. For honey and pollen, a positive association between DWV-A concentration and mortality was observed. High concentrations always resulted in infections, medium ones in 47% of cases and low ones in 20% of cases. No significant difference was observed between the tested products. In commercial honey and pollen, 7.7 × 10^2^–1.8 × 10^5^ and 1.4 × 10^3^–1.3 × 10^4^ DWV-A copies per gram were found, respectively. The results show that DWV-A transmission via hive products is feasible. The risk of introducing novel viruses and/or strains should be considered in trade regulations by including virus analyses for health certificates of hive products

## 1. Introduction

The international trade of honey bee products increased over the past decades, thereby enhancing chances for the spread of bee diseases [1,2]. Indeed, many honey bee pathogens have already been discovered in traded hive products [2]. To protect the health of humans, animals and plants, most countries joined the Terrestrial Animal Health Code (26th edition) released by the World Organization for Animal Health (OIE) in 2017, in which the trade of animal products is also regulated. However, amongst the honey bee pathogens, viruses are not covered by the terrestrial code due to a lack of specific criteria (OIE 2016), even though they are frequently associated with honey bee products and may potentially cause harmful effects [3,4,5]. This seems surprising because it seems most likely that viruses are also spreading as a side effect of the worldwide trade of bee products. Regarding the transmission of viruses, the international movement of live honey bees arguably plays the main role in the global dispersal of bee viruses [6], facilitating the transmission between colonies. Imported bee packages containing viral agents may act as a source of infection for other colonies in the surrounding area (horizontal transmission). The viral particles can be horizontally transmitted in different ways, such as when an infected bee drifts from its own colony to another [7], contact between bees during robbing or while foraging in common food sources [8], and also by human activity, when contaminated material is shared between colonies and apiaries [8]. The international trade of queens, in addition, allows the introduction of viral agents inside the recipient colonies. It has been shown that queens can hold many viruses at the same time and are able to transmit them vertically to their offspring [3,4,9,10,11]. Viruses have also been detected in honey bee products such as honey, pollen and royal jelly freshly extracted from colonies [3,4,5], as well as pollen pellets recently brought to colonies [5]. Moreover, the infectivity of viruses carried by frames containing honey and pollen (bee bread) has also been shown when colonies became infected after receiving such contaminated frames [5]. Still, the role of honey bee hive products for the transmission of viruses in the trade scenario has not been explored in detail.

In addition to the risk of introducing novel viruses, novel strains of already established ones may pose additional threats. Due to genetic recombination, significant changes in the viral genome may occur resulting from the insertion of gene fragments from another viral strain during coinfection of a host cell [12]. Indeed, recombination is increasingly recognized as a major driver of virus evolution [13]. While the high mutation rates observed in RNA viruses often generate deleterious mutations [14], recombination events purge those deleterious mutations [15] and can often result in adaptations for the virus, such as expanding the host range, evasion of host immunity and changes in virulence [16,17].

Although the emergence of recombinants from deformed wing virus (DWV) genotypes were associated with increasing virulence to western honey bees, *Apis mellifera* [18,19,20,21,22,23,24], the potential of genetic recombination is not fully understood in the context of honey bee viruses.

Ubiquitous DWV is amongst the most harmful pathogens of honey bees [6,25,26,27,28]. It can cause clear clinical symptoms, such as crippled wings and a reduced host lifespan [27,29,30], and is a known key driver of honey bee colony losses [31,32]. DWV is a positive sense single-stranded RNA virus (family Iflaviridae; genus Iflavirus) [26,33] and is a recent global epidemic in honey bees [32]. The latter is probably driven by the ectoparasitic mite, *Varroa destructor*, because it is a very efficient vector of DWV. It generates a disease epidemic within the honey bee colony, which then dwindles until it dies [26,34,35]. *V. destructor* have also reduced the genetic diversity of DWV [36], promoting the spread and global distribution of DWV genotype A (DWV-A). The emergence of new genotypes, such as DWV genotype B (DWV-B, also known as Varroa destructor virus-1), has raised concern about their differences in virulence [22,37,38,39]. DWV is infecting honey bees, wild bees and probably other arthropods [5,40] (reviewed in [41]). A better knowledge of the transmission of this particular honey bee virus is therefore of importance, due to the considerable concern for both apiculture and nature conservation efforts.

In this study, we investigate whether or not honey bee hive products can, in principle, act as matrices for DWV-A transmission and how efficient they are. To achieve this goal, fully-crossed laboratory hoarding cage experiments were conducted and complemented with a survey of DWV-A titers in commercial bee products. Since royal jelly and propolis are known to have antimicrobial properties [42], our study focused on honey, pollen and wax.

## 2. Materials and Methods

### 2.1. Study Set Up

In Bern, Switzerland, twelve queenright local honey bee colonies, *A. mellifera*, were screened for DWV-A infections. Adult workers (*N* = 30) were collected from middle frames of each colony in March and April 2015, pooled and tested for DWV-A using RT-qPCR [43]. The three colonies with the lowest infection levels were chosen and tested again in June prior to the experiment, when they had a mean of 1.6 × 10^3^, 3.9 × 10^3^ and 6.1 × 10^3^ virus copies per bee, respectively.

One sealed worker brood frame was taken from each experimental colony and placed in an incubator at 34 °C and 70% relative humidity (RH) until adult emergence [44]. After 48 h, freshly emerged workers were randomly distributed between the experimental hoarding cages. The fully-crossed hoarding cage experiments [44] were conducted from June to August 2015 and designed to test whether or not honey bee products spiked with DWV-A are able to induce an infection in honey bees. To see if the efficacy in DWV-A transmission is different among the tested honey bee products, three treatments with different initial amounts of DWV-A (high, medium and low) were used for each product.

Each series of experiments, corresponding to each honey bee product, consisted of four treatments: three treatments where the honey bee product had been spiked with three different concentrations of DWV-A (5 × 10^9^/5 × 10^8^/5 × 10^6^ copies per ml for honey; 1 × 10^9^/1 × 10^7^/1 × 10^6^ copies per g for pollen; 2.5 × 10^8^/2.5 × 10^6^/2.5 × 10^5^ copies per cm^2^ for wax; Table 1) and a non-spiked treatment with UV-sterilized products as control. All treatments consisted of five repetitions. Each cage was equipped with a honey solution feeder (3 mL syringe), a pollen paste feeder (modified centrifugation tube) and a piece of wax foundation (4 cm^2^). Then, 30 newly emerged workers were introduced into each cage and kept for 10 days at 30 °C and 70% RH in the incubator. Dead individuals were removed from the cages daily, recorded and stored at −20 °C. Honey and pollen consumption was also controlled daily and feeders refilled if required. After two days, the spiked honey and pollen products have been consumed in all cases, resulting in an average consumption of 1 × 10^8^ and 3.3 × 10^7^ copies per bee for high, 1 × 10^7^ and 3.3 × 10^5^ copies per bee for medium and 1 × 10^5^ and 3.3 × 10^4^ copies per bee for low DWV treatment, respectively. Then the feeders were replaced with sterilized food. For the wax product, the spiked piece of wax was available for the entire 10 days. At day 10, all remaining bees were stored at −20 °C.

### 2.2. Cage Experiment

#### 2.2.1. Bee Product Preparation

Only honey and pollen that tested negative for DWV-A by RT-qPCR [43] was used for the experiments. Additionally, these were irradiated with UV light for 120 min. During the UV-treatment, the honey (25 g) was mixed by slowly rotating the honey containing tube each 30 min. A 50% (*w/w*) honey solution was prepared using the UV light-treated honey and Milli-Q water (Millipore Corporation, Billerica, MA, USA). The solution was mixed and stored at −20 °C until used in the experiments.

The pollen grains were crushed to a powder using a stone mortar. The grained pollen was spread on a sheet of paper and irradiated with UV light for 120 min. Every 30 min, the pollen powder was mixed using a sterilized spatula. The pollen paste was prepared with the following proportions: 40% UV-light treated pollen, 50% powder sugar and 10% MilliQ water. The pollen paste was then wrapped into aluminum foil and frozen at −20 °C until usage.

The wax was provided as small pieces of organic wax foundation, which was cut in square pieces with an edge length of 20 mm. The pieces of foundation were displayed on a sheet of paper and each side was irradiated with UV light for 30 min. After irradiation, the wax pieces were stored at −20 °C.

#### 2.2.2. Propagation of DWV-A

DWV-A was propagated using standard methods [45]. Red-eyed worker pupae were microinjected with 2 × 10^7^ virus copies in 2 µL PBS solution (Phosphate Buffered Saline; pH 7.4) between the 2nd and 3rd integuments using a 50 µL micro syringe (Hamilton Microliter™ Syringes, Reno, Nevada, USA) and 30-gauge disposable needles. Pupae were incubated at 30 °C and collected after 6 days. Each pupa was macerated individually in 500 µL PBS and homogenized with 100 µL chloroform using strong vortex. After centrifugation at 13,000 rpm for 10 min, the supernatant was collected and stored at −20 °C. DWV-A was quantified using RT-qPCR [43] and diluted to 1 × 10^7^ copies per µL to make a stock solution for the spiking of the bee products.

#### 2.2.3. Spiking Bee Products with DWV-A

In a pilot study, the average amount of honey and pollen consumed by a single bee per day was estimated. On average, one bee consumed 20+/−3.1 mg of honey solution and 16+/−3.6 mg of pollen paste per day. The DWV-A concentration in the bee products was estimated to apply the required number of virus copies within the first two days of the experiment for honey and pollen.

The honey solution was spiked according to the different DWV-A concentrations, keeping the ratio of honey and water 1:1 (*w/w*). In the case of pollen, the DWV-A solution replaced the water in the recipe for the pollen paste (10%). A homogenous spiking of wax was not possible because the virus RNA would degrade at temperatures where the wax would melt. Therefore, the virus solution was applied on the surface of the pieces of wax foundation and allowed to rest overnight for the water to evaporate. Since bees do not consume wax, the desired virus amount was applied on the piece of wax foundation, which was left in the cage for the entire duration of the experiment.

#### 2.2.4. Cage Construction

The cages were clear polystyrene cups with a diameter of 63 mm and an inner volume of 75 cm^3^ (RIWISA AG Kunststoffwerke, Hägglingen, Switzerland). The cups were turned upside down with the lid acting as the bottom of the cage. Each cage was fitted with three holes for ventilation (large hole in lid covered with a mesh fabric permeable to air) as well as for holding the pollen (2 mL micro-centrifuge tubes) and honey (2 mL plastic syringes) feeders.

#### 2.2.5. Detection of DWV-A

To test for overt virus infection, only bee heads (*N* = 10 per cage) were considered for analyses [46]. The heads were removed using a scalpel sterilized using EtOH and flaming after each cut. Heads from the same treatment were homogenized individually in 100 µL TN-Buffer (10 mM Tris HCl, 10 mM NaCl) using a metal bead (5 mm diameter) and an electronic crushing shaker machine (Retsch Mixer Mill MM 300, Haan, Germany). A NucleoSpin^®^ RNA II kit (Macherey-Nagel, Oensingen, Switzerland) was used for RNA extraction following the manufacturer’s recommendations and using 50 µL of pooled bee head homogenates from the same cage. The purified RNA was then eluated using 60 µL of RNase free water (Macherey-Nagel, Oensingen, Switzerland).

Reverse transcription was performed using standard protocols [32]. The concentration of RNA was measured using a spectrophotometer (Witec NanoDrop^®^ ND 1000 Spectrophotometer, Sursee, Switzerland). Then, 1 µg of RNA and 0.75 µL of 100 mM hexamer primer (Microsynth AG, Balgach, Switzerland) were heated at 70 °C for 5 min and then cooled down to 4 °C. To obtain a final volume of 25 µL, a master mix, consisting of 5 µL M-MLV Reaction Buffer (Promega, Fitchburg, Wisconsin, USA), 1.25 µL 2.5 mM dNTP Mix (Bioline, London, UK) and 1 µL 200 u/µL M-MLV reverse transcriptase (Promega, Fitchburg, Wisconsin, USA), was added and heated to 37 °C for 60 min, before cooling down to 4 °C. A total of 10 fold dilutions were used for the quantification assays.

Each sample was tested for DWV-A using quantitative PCR (qPCR) [32]. The qPCR was conducted using the KAPA SYBR^®^ Fast Universal qPCR kit (KAPA Biosystems, Wilmington, Massachusetts, USA). Briefly, a 12 µL total volume reaction consist of 6 µL of 2x reaction buffer, 0.24 µL forward and reverse primers each (10 μM, Table 2), 2.52 µL water and 3 µL of cDNA. The qPCR was run using an Illumina^®^ Eco Real-Time PCR System (Illumina, San Diego, CA, USA. The amplification conditions were initiated by heating to 95 °C for three minutes in order to activate the polymerase. Then, during each of 40 repeating cycles, the samples were heated to 95 °C for three seconds and cooled down to 57 °C for 30 s. For melting, curve analysis samples were heated to 95 °C for 15 s, cooled down to 55 °C for another 15 s and heated from 55 °C to 95 °C while the strand dissociation was recorded.

Standard curves prepared from DWV-A and *A. mellifera* β-Actin gene were used for virus quantification and normalization, respectively. The standard curve dilutions (10^−2^ to 10^−5^ ng/reaction) were prepared from purified PCR products (Table 2). Two kinds of negative controls were applied (1: RNA-extraction control without bee sample to check for possible contamination in the reagents; 2: PCR negative control, using water instead of cDNA template).

### 2.3. Survey of DWV-A in Commercial Honey and Pollen Products

Honey (*N* = 34) and pollen (*N* = 5) products were acquired from a variety of local Swiss grocery stores. The honeys originated from all continents except Antarctica and the pollen originated from Spain. For extracting RNA from honey and pollen, 120 mg of these bee products and 200 µL of TN-Buffer were mixed thoroughly by using a shaker (Retsch Mixer Mill MM 300, Haan, Germany) and a metal bead (2 mm diameter) that was put inside the sample tube. After shaking, the sample was centrifuged for 5 min at 14,000 rpm. 50 µL of the supernatant were then used for the RNA extraction following the NucleoSpin^®^ RNA II kit (Macherey-Nagel, Oensingen, Switzerland) protocol. Reverse transcription as well as qPCR [32] were conducted as described above.

### 2.4. Statistical Analyses

The statistical analyses were performed using the NCSS statistical software version 10. Since the Kolmogorov-Smirnov test rejected normality in all the used data sets (Test value = 0.289 for virus copies data, 0.305 for delta DWV-A data, 0.214 for cage infection data and 0.246 for mortality data), non-parametric tests were used. The sample size was the number of cages per treatment (*N* = 5) because considering each bee individually would result in pseudo replications [39]. For all tests a critical *p*-value of 0.05 was used.

Using the log rank test with a Bonferroni correction, we checked each bee product experiment for significant differences in mortality between treatments of different DWV-A concentrations. Bees from all five cages underlying the same treatment were integrated for the survival analyses. There were 150 bees per treatment and the survival was recorded for 10 days.

The Kruskal-Wallis multiple-comparison z-value test with Bonferroni correction was used to test for differences in DWV-A titers between high, medium and low DWV-A treatments as well as control treatments with sterilized products. As before, all bees from the same treatment were integrated to one population.

To compare the DWV-A transmission efficacy between different honey bee products a ∆ DWV-A level was calculated. The average number of DWV-A copies taken up by the bees during the experiment was subtracted from the detected DWV-A copies in the heads. Thus the ∆-DWV-A value was a measure for DWV-A replication within the bees. For the analysis, all bees receiving virus copies via the same honey bee product were integrated. In addition, here the Kruskal-Wallis multiple-comparison *z*-value with Bonferroni correction was used.

## 3. Results

### 3.1. Cage Experiment

#### 3.1.1. Survival

**Figure 1:**
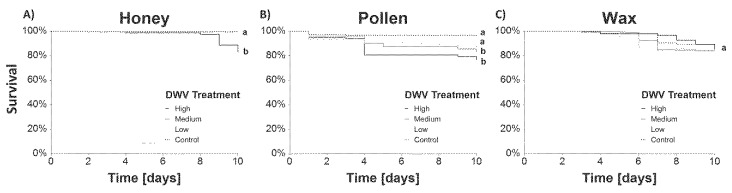
Kaplan-Meier survival plots of caged honey bee workers over the experimental period for each bee product ((**A**) honey, (**B**) pollen and (**C**) wax) with high, medium and low initial DWV-A concentrations. Survival for each treatment was pooled from 5 cages with 30 bees each. Significant differences are indicated by different letters (a,b).

With the exception of one outlier, death rates of less than 15% mortality during the ten-day experiments were observed in all control cages. In the case of the honey assays, the mortality was significantly higher at the high DWV-A treatment compared to all lower DWV-A concentrations and the control (Log Rank test, *p* < 0.0001 per each pair-wise comparison). In the pollen assays, the control cages had significantly lower death rates than those of high (*p* < 0.0001) and medium (*p* = 0.0005) DWV-A treatment. Concerning wax, no significant differences in mortality between different treatments were found (Figure 1).

#### 3.1.2. DWV-A Infection Levels

**Figure 2:**
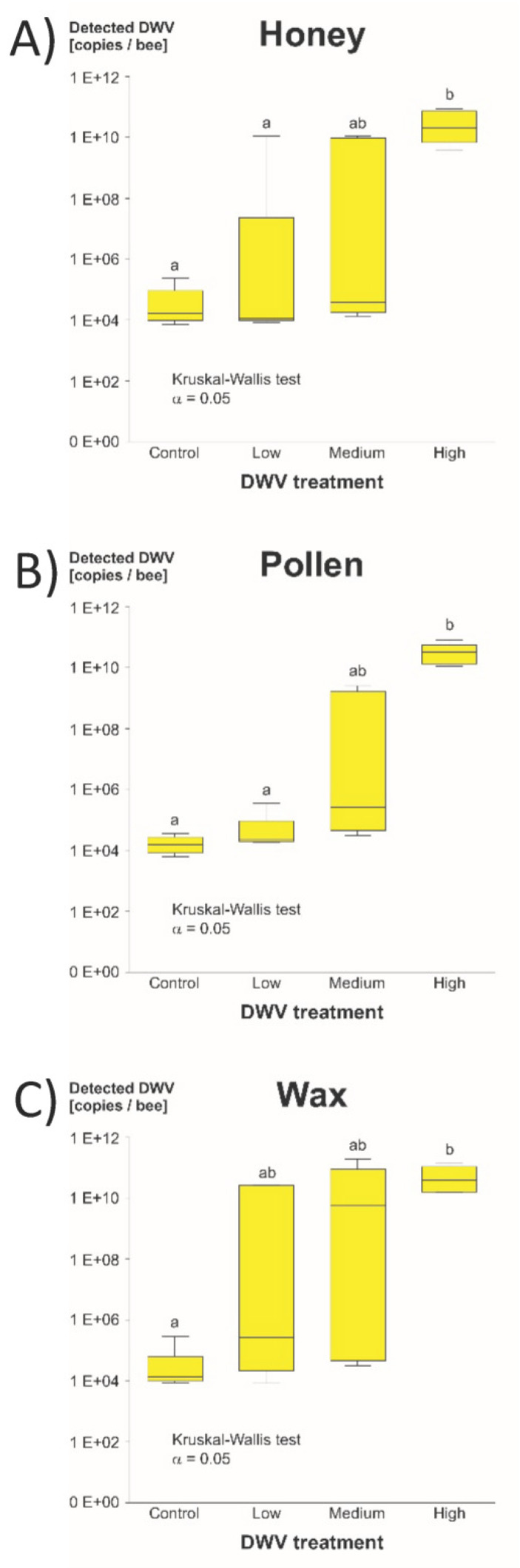
DWV-A copies detected per bee head in the different treatments ((**A**) honey, (**B**) pollen and (**C**) wax). Medians, upper and lower quartiles and maximum and minimum are shown. Significant differences are indicated by different letters (a,b).

While the Kruskal-Wallis test indicated significantly higher DWV-A titers in the heads of bees from high DWV-A treatments compared to bees from control treatments in honey (*z* = 2.83), pollen (*z* = 3.64) and wax (*z* = 2.89), the high DWV-A treatments also showed significantly higher DWV-A titers compared to the low DWV-A treatments in the case of honey (*z* = 2.78) and pollen (z = 2.83) treatments. The critical significance level of the z-value, concerning Bonferroni correction, was 2.64 with the *p*-value set at 0.05. As in the survival curves, the correlation between initial DWV-A concentration and mortality or virus titers, respectively, was the strongest in pollen and the weakest in wax (Figure 2).

**Figure 3:**
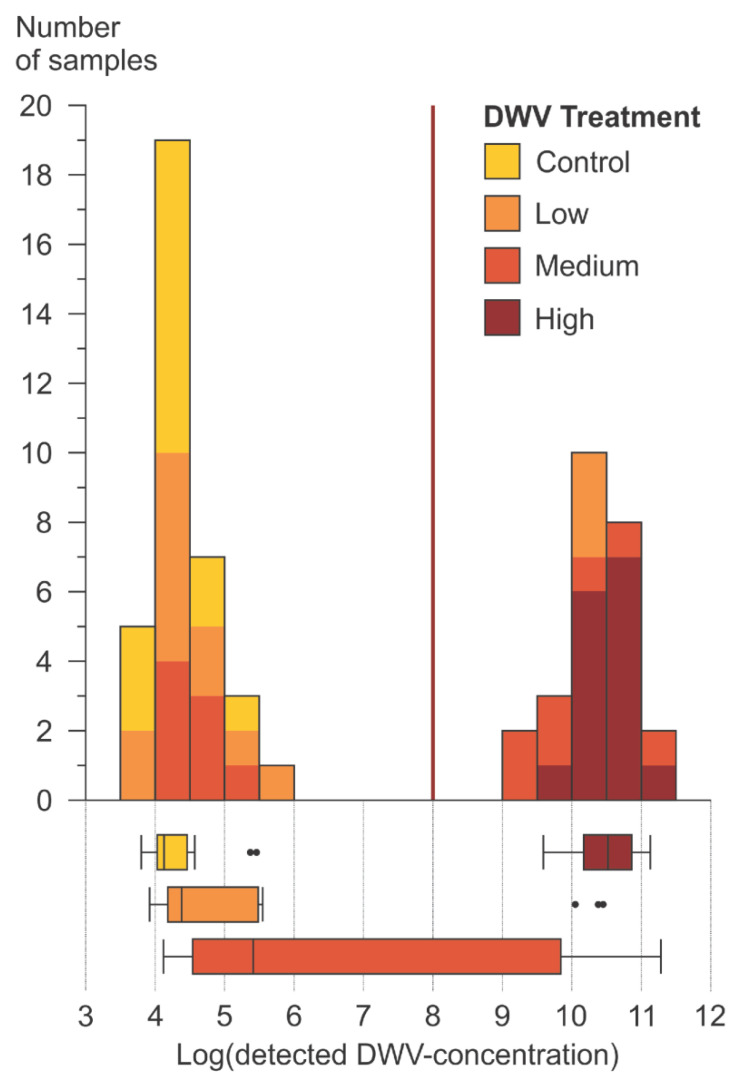
Frequency distribution of DWV-A copy numbers per bee head, all tested hive products pooled together. The highest amount of initially provided DWV-A during the experiment was 1.0 × 10^8^ copies per bee (red line). There is a significant bimodal distribution (Kolmogorov-Smirnov normality, test value = 0.289) with one group showing high DWV-A titers and another group showing low DWV-A titers. The virus titers of the two groups are significantly different (Kruskal-Wallis test, *z*-value = 7.8444 with *p*-value set at 0.05) and there was significantly more virus detected in the high virus titer group than was initially provided during the experiment (*z*-value = 5.6537 with *p*-value set at 0.05). This implies that bees with a high DWV-A titer got infected and that virus replication took place. The box plots at the lower part of the figure show differences between the detected virus titers of different treatments.

According to the detected amount of DWV-A in their heads, a significant bimodal distribution (Kolmogorov-Smirnov normality, test value = 0.289), was found (Figure 3). The group with the lower DWV-A showed between 5 × 10^3^ and 5 × 10^5^ (median = 2.2 × 10^4^) copies per bee, while the group with the higher DWV-A showed between 1 × 10^9^ and 2 × 10^11^ (median = 2.0 × 10^10^) copies per bee. Bees from the higher DWV-A group had significantly more viruses (between one to five orders of magnitude) than the initial fed amount (from 3.3 × 10^4^ to 1 × 10^8^ copies per bee; Kruskal-Wallis test, *z*-value = 5.65). Therefore, virus replication can be considered to occur in those bees.

Regarding the frequency of infection, the number of bee cages that showed an infection as defined above was different depending on the treatment. High DWV-A treatment resulted in an infection of all cages independent of the bee product. At the medium DWV-A treatment, there were two out of five infected cages in honey and pollen each, while there were three in wax. The largest difference was seen at low virus concentrations. There was one infected cage in the case of honey, two in the case of wax while no cages were infected in the case of pollen.

**Figure 4:**
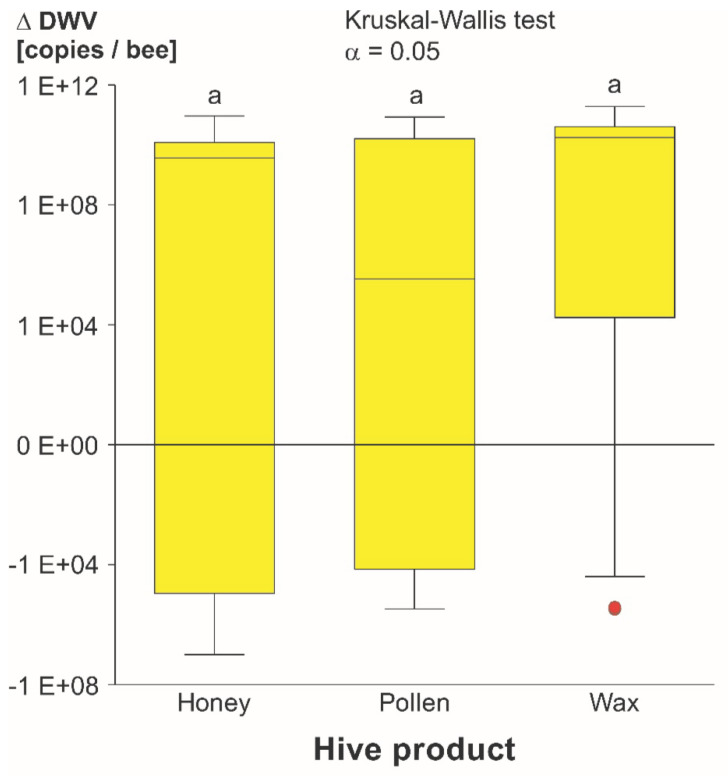
Quantification of replicative DWV-A in bees exposed to DWV-A-spiked honey, pollen and wax. ∆-DWV-A represents the initial amount of DWV-A provided in the products subtracted from the detected DWV-A amount in the bee heads, as a measure for DWV-A replication. For each box, data from high, medium and low initial DWV-A were pooled. There were no significant differences among the bee products.

The comparison between the different bee products showed no significant differences (*z* = 0.38 for honey and pollen, 1.88 for honey and wax and 1.50 for pollen and wax) in virus titers between the different honey bee products (Figure 4).

### 3.2. Survey of DWV-A in Commercial Honey and Pollen

**Figure 5:**
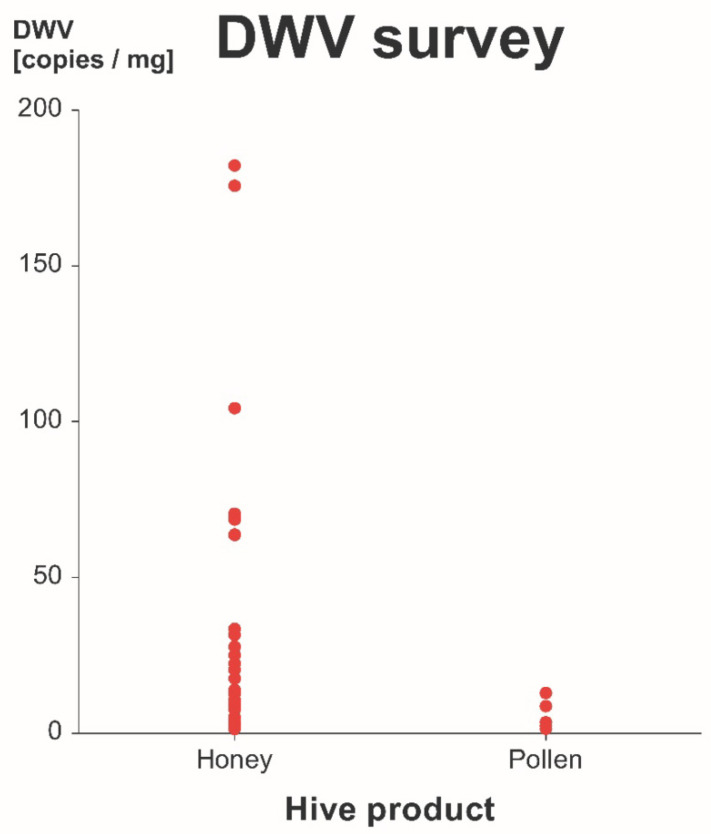
Survey results for commercial honey and pollen. DWV-A copies per mg in 34 commercial honey and 5 pollen samples are shown. Even though a few honey samples showed higher DWV-A titers, no significant difference between the two products was found (Kruskal-Wallis multiple-comparison *z*-value test, *z*-value = 1.9324, *p* > 0.05).

In all honey and pollen samples, the detected amount of DWV-A was low. In honey, between 7.6 × 10^2^ and 1.8 × 10^5^ virus copies with a median of 1.2 × 10^4^ virus copies per gram were detected. In pollen, between 1.4 × 10^3^ and 1.2 × 10^4^ virus copies with a median of 3.5 × 10^3^ virus copies per gram were detected (Figure 5).

## 4. Discussion

Our results clearly show that DWV-A transmission via hive products is feasible. The data also show that mortality increases when honey bees are fed with higher titers of DWV-A via honey and pollen, but not via wax. DWV-A infection was detected in all cages from the high treatment (fed with at least 3.3 × 10^7^ copies per bee), irrespective of the tested bee product. Only very small amounts of DWV-A were detected in the commercial bee products.

### 4.1. DWV-A Transmission Experiment

#### 4.1.1. Survival and DWV-A Titers in Caged Bees

The survival plot shows that only the high DWV-A treatment affects mortality in the honey treatments. Moreover, this treatment resulted in high DWV-A infections in all cages. This suggests an association between mortality and DWV-A infection by oral consumption of high DWV-A titers (1.0 × 10^8^ virus copies per bee) via honey. In contrast, mortality was not different from the controls at the medium and low treatments, despite the occurrence of high DWV-A titers in some of those cages. Similar to honey, the high DWV-A treatment affects mortality in the pollen assays. Moreover, mortality in the medium treatment was also higher than in controls. On the other hand, no significant difference in mortality was found between the low treatment and the control, which is consistent with the low DWV-A titers detected in the bee heads from the pollen low treatment.

In the case of wax assays, no differences in mortality were found among different DWV-A treatments and controls. One possible reason could be that wax does not serve as a food resource, so the oral pathway is somehow different in comparison to honey and pollen.

#### 4.1.2. Data Structure of Detected DWV-A in Bee Heads

Looking at the detected DWV-A titers in the bee heads, there was one pattern that could be observed across all bee products and treatments, including the controls. All bees could be divided into either a high (median = 2.0 × 10^10^ copies per bee) or a low (median = 2.2 × 10^4^ copies per bee) DWV-A titer group (Figure 3). There was a distinct gap between 5 × 10^5^ and 1 × 10^9^ DWV-A copies per bee, with not a single sample in this range. The highest DWV-A amount fed in the experiment was 1 × 10^8^ copies per bee considering that each bee had fed the same (average) amount of spiked bee product. If that assumption is true, all bees from the group with high detected virus titers (>1 × 10^9^ copies/bee) had at least one order of magnitude more virus copies in their heads than the maximal amount of virus that they had taken up during the experiment. Therefore, all bees with a DWV-A amount of at least 1 × 10^9^ copies can be considered as having experienced DWV-A replication and thus an overt infection. In contrast, there were samples in the group of low detected DWV-A titers that showed considerably less DWV-A copies than have been fed, indicating that infection does not always occur. Since up to 5 × 10^5^ DWV-A copies per bee were found even in bees from the negative control, these virus titers are likely to represent DWV-A cover infection levels.

#### 4.1.3. Product Comparison

The honey treatments were spiked with more virus copies compared to pollen and wax, because the different physical properties of the hive products only enabled a certain maximum of treatment solution to be absorbed. Hence, a direct comparison of the tested hive products for viral titers was not feasible. Instead, we compared the DWV-A transmission efficacy between the products using a ∆-DWV-A level as a measure for virus replication within the bees. A significant difference of transmission efficacy between honey, pollen and wax was not found. Even though the obtained results from different bee products are mostly similar, there might be a trend that wax transmits DWV-A easier than honey and pollen. This can be seen in Figure 4, where the median of wax is the highest. That implies that there was more virus replication in the wax treatments, compared to the two other bee products. Indeed, the high levels of the detected virus in the low DWV-A treatment of wax were not significantly different from the high DWV-A treatment, which stands in contrast to the observations of the other products. This might be a clue for a lower DWV-A threshold for an infection via wax. Another indicator can be found when looking at the number of individual cages in which DWV-A replication occurred. In the case of wax, replication took place in two out of five cages in low DWV-A treatment and in three out of five cages in medium DWV-A treatment (number of infected cages: honey low = 1; pollen low = 0; honey medium = 2; pollen medium = 2). However, the impression that wax might be the most efficient matrix of all tested products could also have a methodical reason: in contrast to the honey and pollen assays, where bees consumed the spiked products during 48 h, the spiked pieces of wax were in contact with the bees for 10 days. The amount of virus was similar in the wax compared to the pollen and honey assays, but the virus was highly concentrated on the surface due to an inhomogeneous DWV-A distribution. Apart from that, a different transmission route, such as topical transmission [49], may produce different effects in the host parasite interactions since wax does not serve as food for honey bees.

### 4.2. Survey of DWV-A in Commercial Honey and Pollen

Detected amounts of DWV-A in commercially available honey and pollen were relatively low (1.8 × 10^5^ and 1.2 × 10^4^ copies per gram, the highest values, respectively). However, Mazzei and colleagues [50] found up to 3.0 × 10^6^ virus copies per gram in pollen samples freshly collected by honey bee workers. This amount is 230-fold higher compared to our highest value in pollen. One reason for this difference could be the quick degradation of DWV-A particles at room temperature [51]. In the same line, Graystock and colleagues [52] only detected DWV-A in 2 out of 25 samples of pollen provided as food for bumblebee colonies.

#### Transmission Risk Under Realistic Conditions

Overall, the transmission risk of DWV-A via bee products under realistic conditions is not very high. Nevertheless, it is possible and should be considered. In the case of pollen, the cage experiment shows that a transmission and infection, defined by detection of high DWV-A titers, can only take place if the supplied DWV-A concentration lies between 1 × 10^6^ and 1 × 10^7^ copies per gram. However, in all samples tested in the survey of commercial pollen none reached a concentration in that range. Therefore, the risk of DWV-A to produce infection when transmitted via pollen is considered minimal.

By contrast, honey appears more efficient as a potential matrix for DWV-A transmission. With one out of five cages infected at a concentration of 5 × 10^6^ virus copies per milliliter, a transmission is possible even at lower concentrations. Since we had no test point at lower concentrations, the minimal infectious dose of DWV-A via honey was not clearly defined. However, there were 3 out of the 33 honey samples that showed concentrations of more than 1 × 10^5^ virus copies per milliliter in the commercial honey survey. Thus, those three samples were very close to the concentration that induced infection in one out of five cages. Based on these data, the risk of DWV-A transmission via honey under realistic conditions is 3/33 × 5 which is around 2% with the transmission threshold set at 1 × 10^5^ DWV-A copies per milliliter. It is known that viral particles exposed to environmental conditions (e.g., humidity and heat) may quickly deteriorate [51,53,54]. This variable has not been thoroughly tested in this study. Nevertheless, it has been shown that viruses contained in frames with honey and bee bread remain infective even after been stored by six months at room temperature [5]. Since honey and bee bread stored in frames may represent a different kind of matrices than commercial processed honey and pollen products, data regarding the infectability of DWV in such commercial processed hive products stored for longer periods of time are needed. Until then, even though the estimated infection risk of 2% appears to be rather small, it is only a question of time before an infection occurs, especially if bees consume large amounts of imported honey. Moritz and Erler [55] observed a positive correlation between the amount of honey produced per colony and the amount of honey imports. Thus, they are considering the possibility of relabeling imported honey to locally produced honey. This illegal relabeling might be disguised by feeding the imported honey to local bee colonies. In that case, local bees would consume huge amounts of imported and possibly contaminated honey, which would increase the chance of infection dramatically.

For wax as a matrix, we have no data for realistic DWV-A concentrations of commercial products. That makes it impossible to evaluate a realistic transmission risk. However, wax is often heated during commercial processing and this may inactivate viruses, thereby decreasing the role of wax foundation as a virus carrier in the global trade scenario. By contrast, wax showed some transmission potential in the cage experiments and should thus also be considered as a potentially effective DWV-A vehicle, e.g., as a result of the common apicultural practice of exchanging non-processed combs between colonies.

As DWV is ubiquitous in most continents, a major concern should be the introduction of novel, potentially more virulent strains. Genetic recombination between different strains may indeed result in new viral variants with enhanced virulence. For example, distribution of DWV-B (also known as VDV-1) has expanded over the last decade [56] and recombination events with DWV-A appear to be frequent [18,19,20,21,22,23]. Moreover, the selection of a virulent recombinant variant between DWV-A and DWV-B has been already reported [23]. However, due to the potent role of *V. destructor* as a virus vector [35], it appears as if a recombinant DWV variant with lower virulence has been selected to optimize its transmission by *V. destructor* [57]. Therefore, more studies are necessary to assess the real impact of DWV genetic recombination on the emergence of potentially more virulent strains.

Risk is composed of the probability of occurrence and the damage caused if that undesired event actually occurs [58]. Therefore, the potential of hive products for virus transmission reported here should be considered, even though the probability of occurrence is probably rather low. Indeed, the potential damage resulting from novel viral strains could obviously be severe. Therefore, the potential of hive products for virus transmission should be considered in daily beekeeping practice. For example, it appears very risky to feed cheap honey to colonies, which is unfortunately a common practice of some beekeepers. A sustainable way to limit the risk effectively would be to include virus analyses in health certificates for import and export of hive products.

## 5. Conclusions

Our data clearly show that transmission of DWV-A via hive products is feasible. Since there are currently no regulations aiming to limit the spread of viruses due to global trade, the results are calling for respective mitigation measures, i.e., health certificates for hive products.

## Figures and Tables

**Table 1:** Initial Deformed wing virus genotype A (DWV-A) genome copy numbers in the different treatments. The number of DWV-A copies per contaminated honey, pollen and wax are given. For comparison, the average number of DWV-A genome copies consumed per bee individual (virus copies per cage divided by number of bees in cage) is also listed.

Treatment	Hive Product		
Group	Honey	Pollen	Wax
High (per mL/g/cm^2^)	5.0 × 10^9^	1.0 × 10^9^	2.5 × 10^8^
High (per bee)	1.0 × 10^8^	3.3 × 10^7^	3.3 × 10^7^
Medium (per mL/g/cm^2^)	5.0 × 10^8^	1.0 × 10^7^	2.5 × 10^6^
Medium (per bee)	1.0 × 10^7^	3.3 × 10^5^	3.3 × 10^5^
Low (per mL/g/cm^2^)	5.0 × 10^6^	1.0 × 10^6^	2.5 × 10^5^
Low (per bee)	1.0 × 10^5^	3.3 × 10^4^	3.3 × 10^4^

**Table 2:** PCR primers used for the relative virus quantification of DWV-A.

Target	Sequence (5′–3′)	[bp]	Ref
DWV-A	TTC ATT AAA GCC ACC TGG AAC ATC	136	[47]
	TTT CCT CAT TAA GTG TGT CGT TGA		
β-actin	CGT TGT CCC GAG GCT CTT T	66	[48]
	TGT CTC ATG AAT ACC GCA AGC		
